# Prevalence and Correlates of Neurocognitive Disorders among HIV Patients on Antiretroviral Therapy at a Kenyan Hospital

**DOI:** 10.1155/2019/5173289

**Published:** 2019-10-30

**Authors:** A. G. Mugendi, M. N. Kubo, D. G. Nyamu, L. M. Mwaniki, S. K. Wahome, J. E. Haberer

**Affiliations:** ^1^Department of Pharmaceutics and Pharmacy Practice, School of Pharmacy, University of Nairobi, Nairobi, Kenya; ^2^Department of Clinical Medicine and Therapeutics, School of Medicine, University of Nairobi, Nairobi, Kenya; ^3^Christian Health Association of Kenya, Nairobi, Kenya; ^4^Kenyatta National Hospital, Nairobi, Kenya; ^5^Massachusetts General Hospital and Harvard Medical School, Boston, MA, USA

## Abstract

**Background:**

HIV-associated neurocognitive disorders (HAND) represent a spectrum of cognitive abnormalities affecting attention, concentration, learning, memory, executive function, psychomotor speed, and/or dexterity. Our objectives in this analysis are to determine the prevalence of HAND and the covariates in a Kenyan population.

**Methods:**

We conducted a cross-sectional study in a convenient sample of people living with HIV on antiretroviral therapy (ART) attending routine care visits at the Kenyatta National Hospital HIV clinic between July and August 2015. Baseline demographics were obtained using interviewer-administered questionnaires; clinical data were abstracted from patient records. Trained research clinicians determined the neurocognitive status by administration of the International HIV Dementia Scale (IHDS), the Montreal Cognitive Assessment (MOCA) scale, and the Lawton Instrumental Activities of Daily Living (IADL) scale. Cognitive impairment was defined as a score of ≤26 on the MOCA and ≤10 on the IHDS. Descriptive analysis and logistic regression to determine predictors of screening positive for HAND were done with the significance value set at <0.05.

**Results:**

We enrolled 345 participants (202 men; 143 women). The mean age of the study population was 42 years (±standard deviation (SD) 9.5). Mean duration since HIV diagnosis and mean duration on ART were 6.3 (±SD 3.7) and 5.6 years (±SD 3.4), respectively. Median CD4 count at interview was 446 cells/mm^3^ (interquartile range (IQR) 278–596). Eighty-eight percent of participants screened positive for HAND, of whom 87% had asymptomatic neurocognitive impairment (ANI) and minor neurocognitive disorders (MND) grouped together while 1% had HIV-associated dementia (HAD). Patients on AZT/3TC/EFV were 3.7 times more likely to have HAND (OR = 3.7, *p*=0.03) compared to other HAART regimens. In the adjusted analysis, women were more likely to suffer any form of HAND than men (aOR = 2.17, 95% CI: 1.02, 4.71; *p*=0.045), whereas more years in school and a higher CD4 count (aOR = 0.58, 95% CI: 0.38, 0.88; *p*=0.012), (aOR = 0.998, 95% CI 0.997, 0.999; *p*=0.013) conferred a lowered risk.

**Conclusion:**

Asymptomatic and mild neurocognitive impairment is prevalent among people living with HIV on treatment. Clinical care for HIV-positive patients should involve regular screening for neurocognitive disorders while prioritizing women and those with low education and/or low CD4 counts.

## 1. Introduction

The brain is the second most affected organ by HIV infection (the lungs are the most affected organ) [1]. Uncontrolled viral replication within neural tissue results in a chronic inflammatory state that can present as behavioral, motor, and/or cognitive abnormalities [2]. These abnormalities, called HIV-associated neurocognitive disorders (HAND), can lower the quality of life among persons living with HIV by interfering with activities of daily living such as employment and importantly adherence to prescribed antiretroviral therapy and other medicines [3].

The introduction of highly active antiretroviral therapy (HAART) significantly lowered the prevalence of HAND but did not eliminate the disorders [4–6]. There is a paucity of data on the prevalence of these disorders in the Kenyan population, primarily due to a lack of routine screening in scheduled clinic visits.

Several risk factors for HAND are documented in the literature from different populations. Age is a well-established covariate for the progression of HIV. Others include female sex, stage of HIV disease, comorbidities such as hepatitis B and C, intravenous drug use, lower educational achievement, low CD4 count (especially ≤200 cells/*μ*L), and low hemoglobin concentration [7–9]. These risk factors may or may not apply to the Kenyan population due to varied demographics and heterogeneity in patterns of disease progression, as well as differences in the HIV subtypes and clades that are predominant in the region.

To determine the burden of HAND among people living with HIV on HAART in Kenya and identify the risk factors for these disorders, we conducted a cross-sectional study involving neurocognitive function assessment in a routine HIV care clinic.

## 2. Methods

### 2.1. Study Design

We conducted a cross-sectional study at the HIV clinic of the Kenyatta National Hospital in Nairobi, Kenya between July and August of 2015. The clinic offers care to persons living with HIV—children, adults, and pregnant women—drawn from Nairobi County and its environs. It is open five days a week and is run by a team of clinicians comprising medical doctors, pharmacists, nurses, and other cadres (counselors, social workers, peer educators, and psychologists).

Patients with clinic dates scheduled on the study days were targeted for recruitment using convenience sampling; recruitment was done by a registered nurse during triage as patients arrived for their scheduled clinic visit. Patients were screened using an eligibility checklist, and those who satisfied the following criteria were enrolled consecutively. Eligibility included having a documented HIV-positive status, enrolment for follow-up at the clinic, and being 18 years or older. Patients were excluded if they had history of traumatic brain injury, psychiatric illness, chronic renal failure, chronic liver disease, malignancy, history of substance abuse or alcoholism, and active or known past central nervous system opportunistic infection, all of which may complicate HAND. Informed consent was obtained prior to enrolment.

A target sample size of 329 was estimated using the Fisher et al.'s formula with HAND prevalence data from a Ugandan study [10]. We enrolled 5% greater than the target sample size to cater for potential missing data. Research staff administered standardized case report forms and did chart reviews to collect data on demographics and clinical history. Research clinicians, who were recruited from among the clinic staff, were trained to use the Montreal Cognitive Assessment (MOCA) scale, the International HIV Dementia Scale (IHDS), and the Lawton Instrumental Activities of Daily Living (IADL) scale to assess neurocognitive functioning and administered them during participant interviews.

The MOCA is a quick and easy-to-use screening tool for neurocognitive impairment that consists of 13 tasks that measure eight cognitive domains, namely, attention, language, abstraction, delayed recall, visual spatial/executive, naming, memory, and orientation. It can be administered in 10–15 minutes, and summing up the individual scores gives a total score. The maximum score is 30 points; a score of >26 is considered normal, while a score ≤26 is indicative of cognitive impairment. For participants with less than 12 years of formal education, one point was added to the total score [11].

The IHDS consists of three subtests: timed finger tapping, timed alternating hand sequence test, and recall of four items at 2 minutes. The maximum total score is 12 with a contribution of 4 points from each subtest. A participant who scores ≤10 should be evaluated for possible cognitive impairment [10].

The IADL was used to assess functional status and was primarily designed to assess a person's ability to live independently. Eight domains are measured using the IADL: ability to use a telephone, shopping, food preparation, housekeeping, laundry, mode of transportation, responsibility for own medication, and ability to handle finances. Participants were scored by choosing the item description that most closely resembled their highest functional status (either a 0 or a 1), and the summary score ranged from 0 (low function, dependent) to 8 (high function, independent) [12].

The three tests are complementary; the MOCA assesses multiple cognitive domains, the IHDS adds a motor domain and an additional assessment of memory, whereas the IADL provides an assessment of functionality. HAND was categorized based on the joint scores on the MOCA and IHDS. The IADL was to provide a distinction between asymptomatic neurocognitive impairment (ANI), mild neurocognitive disorder (MND), and HIV-associated dementia (HAD). The Frascati criteria were applied to determine the category of HAND based on the means of the aggregate scores of the MOCA and IHDS and also considering the IADL score [13].

Ethical approval to conduct the study was obtained from the Kenyatta National Hospital, University of Nairobi Ethics and Research Review Committee (reference KNH-ERC/1/136).

### 2.2. Data Analysis

A database was created using Epi Info (version 7, CDC, Atlanta, GA, USA), and the data that had been collected on hard copy case report forms were transferred therein. Statistical analysis was performed in STATA (version 13, College Station, TX, USA). Categorical variables were detailed in frequency tables, and continuous measures were summarized using means and standard deviations or medians and ranges, as appropriate. After assessing for normality with the Shapiro–Wilk test and plotting histograms and QQ plots, we used the *T*-test to check for differences in mean values of continuous variables and chi-square tests for binary variables.

To determine the covariates for prevalence of HAND (defined as a positive screen for cognitive impairment on both the MOCA and IHDS), we performed logistic regression modelling. We used backward stepwise selection to identify parameters to fit in the final model. The selected model was run with an interaction term for age and gender. The threshold for statistical significance was set at *α* = 0.05.

## 3. Results

Three hundred and forty-five participants were recruited, the majority of whom were women (*n* = 202, 59%; Table 1). Their mean age was 42 years (SD ± 9.5). Nearly three quarters of the participants had attained a high school education. The median CD4 count at enrolment was 446 cells/mm^3^ (IQR 278–596). Among the 53 participants whose viral load assays were available, 53% were virally suppressed (Table 2). The most commonly prescribed HAART regimen was a combination of tenofovir, lamivudine, and efavirenz, which is in line with the national guidelines on the management of HIV infection [14].

Using the IHDS, 302 participants had scores of ≤10 (Table 3). The mean scores for patients with and without possible dementia were 8.22 (±0.09) and 11.20 (±0.06), respectively, which were statistically significantly different (*t* statistic −27.24, MD = −2.97 (0.11), 95% CI: (−3.18, −2.75), *p* < 0.0001).

With the MOCA, 289 participants had scores of ≤26 (Table 4). The mean score was 19.7 (±4.8). The mean scores classified as normal and abnormal were 26.98 (0.13) and 18.57 (0.24), respectively. These scores were significantly different (*t* statistic 30.75, MD = 8.41 (0.27), 95% CI: (19.23, 20.26), *p* < 0.0001).

Application of the IADL to 345 participants indicated that 344 were functionally independent, whereas only one had some mild dependence. The median score was 8 (IQR 5–8) with a mean of 7.99 (±0.16). The results of this tool could therefore not be used to apply the Frascati criteria.

**Figure 1:**
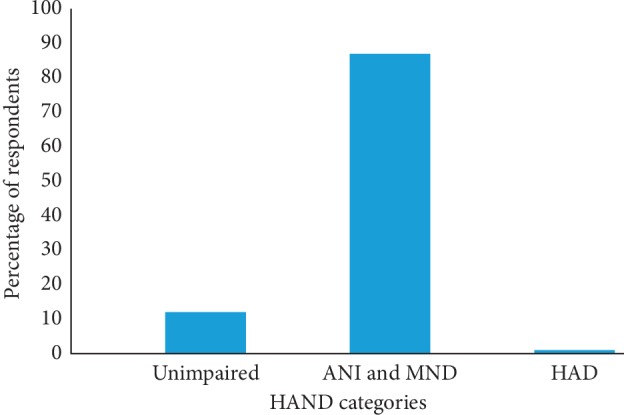
Prevalence of subtypes of HAND among the participants. ANI: asymptomatic neurocognitive impairment; HAD: HIV-associated dementia; MND: mild neurocognitive dementia.

Based on the MOCA and the IHDS, symptomatic HAND was identified in 6 (1%) of the participants, which was classified as HIV-associated dementia (HAD) (*n* = 6; 1%). Eighty-seven percent of the participants (*n* = 298) had either asymptomatic neurocognitive impairment (ANI) or mild neurocognitive disorder (MND) grouped together. Forty-one (12%) participants did not suffer any form of cognitive impairment (Figure 1).

On bivariate analysis, education and occupation were the only factors at baseline that were correlated with HAND (Tables 5 and 6). Statistically significant associations with any degree of HAND on multivariate logistic regression analysis included increasing level of education (aOR = 0.58) which lowered the risk of HAND by 42%, female sex (aOR = 2.17) showing that women were at a higher risk of HAND compared to men by 117%, and increasing CD4 counts (aOR = 0.998) which conferred a lowered risk by 0.2% (Table 7). Factors associated with the advanced forms of HAND (MND and HAD) were an increasing educational achievement (aOR = 0.24), meaning that patients with higher levels of education had a lowered risk of HAND by 76%, possible depression (aOR = 7.47) and female sex (aOR = 5.83) which were associated with a more than seven-fold and five-fold risk of HAND, respectively (Table 8).

The interaction term between age and gender was not significantly associated with a diagnosis of HAND (aOR 1.01, *p*=0.80), and this lack of an association still held for the more advanced forms of HAND (aOR 1.08, *p*=0.31).

Patients on AZT/3TC/EFV were 3.7 times more likely to have HAND (OR = 3.7, *p*=0.03) compared to other HAART regimens. TDF/3TC/EFV, the most commonly prescribed regimen, was not associated with having any degree of HAND (OR = 1.1, 95% *p*=0.732).

## 4. Discussion

In this cross-sectional study involving PLWH on HAART at a routine care HIV clinic, a majority of participants screened positive for HAND. Approximately 87% of the participants had either ANI or MND grouped together, whereas only 1% had possible HAD. We identified education, gender, and CD4 count as being significantly associated with a diagnosis of possible HAND.

The prevalence of HAND reported in this study is in keeping with other findings from the developing world, where the burden is reported to lie between 14% and 64% [15]. Recent prevalence reports from Uganda, Ethiopia, and West Africa have quantified the burden of HAND to lie between 31–78%, 33–36%, and 21–73%, respectively [7, 10, 16–20]. In countries that lie in the south of Africa, for example Malawi, the prevalence of MND and HAD was 12% and 3%, respectively, similar to our findings [8, 21]. However, in South Africa and Zambia, the burden of both MND and HAD was much higher compared to what we report (42.4% and 25.4% in South Africa and 13% and 19% in Zambia, respectively) [22, 23].

The differences in the prevalence of HAND within Africa could be due to the regional variations in the clades of HIV [2]. Different HIV clades have been demonstrated to harbor varying neuropathogenic potentials. In Uganda, the predominant clades are A and D, the latter having a higher potential of causing HAND. A greater prevalence of HAND was observed in individuals infected with clade D strains compared to those infected with clade A (89 vs. 24%), suggesting a higher virulence with clade D. In West Africa, clades A and G are more prevalent and are less virulent [16]. The most common HIV clade associated with nearly 50% of infections worldwide is C. Studies in China, India, and Botswana have all produced conflicting results regarding the association between clade C and HAND, and therefore, more studies may be required to ascertain this association [2].

Another possible explanation for the differences in prevalence could be the lack of uniformity of the tools used to screen for HAND, as well as the quality of training offered to study staff in application of those tools. Though most used the IHDS, many used several multidomain neuropsychological tests such as the Weschler Memory Scale III (WMS-III), Grooved Pegboard Dominant Hand and Nondominant Hand Test, Instrumental Activities of Daily Living, Verbal Fluency, and Controlled Oral Word Association Test (COWAT) among others.

The introduction of HAART reduced the prevalence of HAND possibly by reducing the deleterious effects of uncontrolled viral replication [2, 6]. Reports indicate that in the pre-HAART era, HAD burden was as high as 16%, but has since fallen to about 5% on average [6]. Although we do not have pre-HAART data, our findings (HAD 1%) and those in other diverse settings such as Japan (HAD 1%) and Malawi (HAD 3%) similarly find a low prevalence of HAD [8, 24].

However, despite a reduction in the prevalence of HAD in the era of HAART, milder HIV-associated neurocognitive dysfunction in the form of MND and ANI still persists [5]. This is despite the use of highly efficacious ART regimens, some of which have high CNS penetration scores that should lead to suppression of CNS HIV replication and associated neuroinflammation. Postulated hypotheses point towards a possible role of the ARV drugs themselves in neurotoxicity.

In our study, for example, patients on AZT/3TC/EFV were 3.7 times more likely to have HAND (OR = 3.7, *p*=0.03) compared to other HAART regimens. Both zidovudine and efavirenz have previously been documented to have possible cytotoxic effects on CNS endothelial cells via increasing oxidative stress and potentiating mitochondrial dysfunction [25, 26]. In the presence of HIV proteins, these ART cytotoxic effects could be further worsened, with altered gene expression as well as activation of inflammatory cell-signaling cascades leading to neuronal cellular dysfunction and apoptosis [25].

Furthermore, a metabolite of efavirenz, 8-hydroxy efavirenz, has been identified as a potent neurotoxin that may damage neuronal dendritic spines in *in vitro* studies and may contribute towards the neuronal damage underlying HAND [27]. Several studies including a multicentre study in the US and another one in Italy have previously reported an association between the use of efavirenz-based ART and HAND [28, 29]. As such, there should be concerted efforts to ensure access to less neurotoxic ARVs in sub-Saharan Africa, particularly among women.

We found that the level of education was significantly associated with the risk of any degree of HAND as well as the advanced forms of the condition (HAD). Each additional year of formal education conferred a 42% reduction in the risk of HAND, which is in agreement with findings from a study in the United States among persons living with HIV [30]. Data on the relationship between HAND and the level of education, however, is mixed. Tsegaw et al. and Joska et al. in Ethiopia and South Africa, respectively, reported that fewer years of formal education are associated with HAND [19, 22], while others have demonstrated no such association [17].

However, in contrast, the sCReen for Anxiety, depression, and Neurocognitive Impairment in HIV-positive patients (CRANIum) study reported that years of education had no association with the risk of HAND [31]. CRANIum was a multinational, multicenter, cross-sectional study conducted in Western Europe and Canada from October 2010 to June 2011 describing and comparing the prevalence of a positive screen for neurocognitive impairment among other things in persons living with HIV either on or naïve to HAART. In this study, the brief neurocognitive screen (BNCS) was applied to check for cognitive impairment, which was different from the tools that we used and could have contributed to the variability observed.

Age, gender, and marital status have not been associated with HAND in patients from diverse settings within sub-Saharan Africa, although our findings did indicate that females were twice as likely to suffer from HAND as males. However, one report from South Africa identified males as being at a higher risk of HAD [22], while others from Japanese and Ethiopian populations reported that older age was associated with an increased risk for HAND [19, 24].

Low CD4 count has been associated with HAND from several studies within sub-Saharan Africa [19, 20, 22]. We similarly found that a higher CD4 count was protective against HAND and reduced the risk by 0.2%.

More advanced forms of HAND (MND and HAD) were also associated with the level of education, with each additional year in school reducing the risk of HAND by 76%, which is in agreement with reports cited in the foregoing paragraphs. Women were nearly six times as likely as men to suffer the severe forms of HAND (aOR 5.83).

Our results illustrate that a large proportion of patients on HAART are asymptomatic, presenting a clinical and ethical dilemma for both the clinician and patient in making a diagnosis that seems to be of no immediate consequence. Indeed, routine screening for these disorders is the exception rather than the rule, not only in our setting, but in other regions as well [32]. However, we aver that a prioritized screening for patients with the identified risk factors may prove more beneficial in the long run, both for the patient by taking steps to retard the disease progression and for the healthcare system by minimizing the costs associated with managing more advanced forms of the disease.

Our study had several limitations. First, due to constrained resources, we used the IHDS and MOCA screening tools as opposed to a comprehensive neuropsychological test battery. According to published criteria, a comprehensive neuropsychological evaluation, including ruling out other CNS causes of cognitive dysfunction, e.g., other CNS infections, CNS tumor, and cerebrovascular disease, remains the accepted standard for evaluation of HAND [33]. However, a 2013 consensus statement from the Mind Exchange Working Group of experts recognizes that in areas with limited resources, a presumptive clinical diagnosis of HAND may be made on the basis of screening tools like the ones we applied, symptom questionnaires, functional assessments, and limited neuropsychological testing [34].

In the future, confounding variables could be better excluded through concomitant administration of these tools and neuroimaging plus examination of the cerebrospinal fluid for markers of inflammation, ARV concentrations to correlate with central nervous system penetration score, and concentrations of B amyloid proteins [2].

A second limitation to our study is the fact that we did not employ demographically corrected norms for our population. Performance on neurocognitive tests has been shown to be influenced by age, education level, ethnic background/race, and gender; thus, neurocognitive tests should be appropriately normed for the study population [33]. In Malaysia, for example, use of the MOCA screening tool using a cutoff of ≤26 considerably overestimated cognitive impairment among HIV-positive patients (69.3%), compared to an impairment rate of 23.4% when norms corrected for age, sex, education, and ethnicity were employed [35]. In mild cognitive impairment, the MOCA screening tool may be particularly sensitive to the effects of education level [36]. As such, our results may have overestimated the neurocognitive impairment rate in our population with widely differing education levels, particularly for mild neurocognitive dysfunction.

Thirdly, application of the IADL did not provide a distinction between ANI and MND. This meant that we could only use the MOCA and the IHDS to partly apply the Frascati criteria resulting in grouping of ANI or MND patients together. Despite this, it is evident that there is some continued degree of CNS damage due to HIV in the presence of ART.

Finally, we could not establish causality and could only make inferences about associations. Despite these limitations, ours is, to the best of our knowledge, the first study to determine the burden of HAND among adult patients on HAART in Kenya.

In summary, we found a high prevalence of HAND, particularly ANI. Years of formal education, female gender, and CD4 counts were found to be associated with HAND. In addition, women with lower educational achievement are at a higher risk for the severe forms of HAND. Future studies in this population should examine the association between ARV adherence and HAND. Recent data suggest that decreased adherence is associated with an increased risk of HAND [37]. In addition, a prospective study to determine the clinical course of HAND would be informative.

## Figures and Tables

**Table 1:** Baseline sociodemographic characteristics of participants.

Characteristic	*n*	%
Sex		
Male	143	41
Female	202	59
Marital status		
Single	79	23
Married	205	59
Separated	14	4
Divorced	9	2
Widowed	38	11
Occupation		
Unemployed	29	8
Self-employed	193	56
Employed	120	35
Student	3	1
Smoking status		
Smoker	10	3
Nonsmoker	335	97
Consumes alcohol		
Yes	34	10
No	311	90

**Table 2:** Baseline clinical characteristics of the participants.

Characteristic	*N*	% or mean (SD)
Hypertensive		
Yes	45	13
No	300	87
Diabetic		
Yes	14	4
No	331	96
CD4 count		
<250	74	22
250–349	49	14
350–499	86	25
≥500	132	38
Missing	4	1
Viral load (*n* = 53)		
Suppressed (<400 copies/ml)	28	53
Unsuppressed (≥400)	25	47
Mean months since CD4 was done	341	15 (13)
Mean years since last viral load	53	1.9 (1.3)
Mean years since HIV diagnosis	345	6.3 (3.7)
Mean years (SD) on ART	345	5.6 (3.4)

**Table 3:** Test domains of the Montreal Cognitive Assessment scale.

MOCA scale	Points/score	Normal *N* (%)	Cognitive impairment *N* (%)	Missing *N* (%)	Total *N* (%)
Visual spatial/executive					
	0/5	0	44 (15)	1 (11)	45 (13)
	1/5	0	30 (10)	—	30 (9)
	2/5	0	42 (15)	1 (11)	43 (13)
	3/5	1 (2)	75 (26)	2 (22)	78 (21)
	4/5	14 (30)	57 (20)	3 (33)	74 (21)
	5/5	32 (68)	41 (14)	2 (22)	75 (22)

Naming					
	0/3	0	3	—	3 (1)
	1/3	0	16 (6)	1 (11)	17 (5)
	2/3	3 (6)	96 (33)	2 (22)	101 (29)
	3/3	44 (94)	174 (60)	6 (67)	224 (65)

Attention digits					
	0/2	0	40 (14)	2 (22)	42 (12)
	1/2	3 (6)	114 (39)	1 (11)	118 (34)
	2/2	44 (94)	135 (47)	6 (6)	185 (54)
Attention letters					
	0/1	1 (2)	59 (20)	1 (11)	61 (18)
	1/1	46 (98)	230 (80)	7 (78)	283 (82)

Attention subtraction					
	0/3	0	26 (9)	2 (22)	28 (8)
	1/3	1 (2)	129 (45)	1 (11)	131 (38)
	2/3	11 (23)	64 (22)	2 (22)	77 (22)
	3/3	35 (74)	70 (24)	4 (44)	109 (32)

Language repeat					
	0/2	1 (2)	124 (43)	3 (33)	128 (37)
	1/2	13 (28)	127 (44)	3 (33)	143 (41)
	2/2	33 (70)	38 (13)	2 (22)	73 (21)
Language fluency					
	0/1	22 (47)	208 (72)	6 (67)	236 (68)
	1/1	25 (53)	81 (28)	2 (22)	108 (31)

Abstraction					
	0/2	3 (6)	140 (48)	—	143 (41)
	2/2	10 (21)	106 (37)	1 (11)	117 (34)
	2/2	34 (72)	43 (15)	2 (22)	79 (23)
	Missing			6 (67)	6 (2)

Delayed recall					
	0/5	0	57 (20)	—	57 (17)
	1/5	0	38 (13)	1 (11)	39 (11)
	2/5	1 (2)	80 (28)	1 (11)	82 (24)
	3/5	15 (32)	62 (21)	2 (22)	79 (23)
	4/5	18 (38)	49 (17)	4 (44)	71 (21)
	5/5	13 (28)	3 (1)		16 (5)

Orientation					
	0/6	0	0	0	0
	1/6	0	0	0	0
	2/6	0	0	0	0
	3/6	0	1 (<1)	0	1 (<1)
	4/6	0	2 (1)	0	2 (1)
	5/6	2 (4)	13 (5)	3 (33)	18 (5)
	6/6	45 (96)	273 (94)	6 (67)	324 (94)

**Table 4:** Test domains of the international HIV Dementia Scale.

International HIV Dementia Scale	Score	Normal *N* (%)	Cognitive impairment *N* (%)	Total *N* (%)
Motor speed				
	15 in 5 seconds	26 (63)	11 (4)	37 (11)
	11–14 in 5 seconds	15 (37)	113 (37)	128 (37)
	7–10 in 5 seconds	0	152 (50)	152 (44)
	3–6 in 5 seconds	0	24 (8)	24 (7)
	0–2 in 5 seconds	0	2 (1)	2 (1)
	Total	**41**	**302**	**343**

Psychomotor speed				
	4 sequences in 10 seconds	26 (63)	41 (14)	67 (20)
	3 sequences in 10 seconds	15 (37)	128 (42)	143 (41)
	2 sequences in 10 seconds	0	92 (30)	92 (27)
	1 sequence in 10 seconds	0	10 (3)	10 (3)
	Unable to perform	0	31 (10)	31 (9)
	Total	**41**	**302**	**343**

Memory recall				
	1.0	0	4 (1)	4 (1)
	1.5	0	4 (1)	4 (1)
	2.0	0	41 (14)	41 (12)
	2.5	0	6 (2)	6 (2)
	3.0	3 (7)	60 (20)	63 (19)
	3.5	0	8 (3)	8 (2)
	4.0	38 (93)	179 (59)	217 (63)
	Total	**41**	**302**	**343**

**Table 5:** Correlation of baseline demographics and HAND on bivariate analysis.

Variable	HAND category				*p* value	*Χ* ^2^
	Unimpaired *n* (%)	ANI *n* (%)	MND *n* (%)	HAD *n* (%)		
Age, years					0.481	4.6
20–29	2 (5)	22 (9)	6 (15)			
30–39	16 (40)	76 (30)	7 (18)	2 (33)		
40–49	19 (48)	108 (42)	16 (41)	2 (33)		
≥50	3 (8)	49 (19)	10 (25)	2 (33)		
Gender					0.193	1.7
Male	21 (51)	107 (41)	14 (35)	1 (17)		
Female	20 (49)	151 (59)	26 (65)	5 (83)		
Education					**0.001** ^*∗*^	**13.7**
Primary	3 (7)	61 (24)	15 (37)	4 (67)		
Secondary	17 (42)	125 (48)	21 (53)	2 (33)		
Tertiary	21 (51)	72 (28)	4 (10)			
Living children					0.022	7.6023
0	3 (7)	21 (8)	5 (13)	0		
1-2	23 (56)	139 (54)	12 (30)	3 (50)		
≥3	15 (37)	97 (38)	23 (57)	3 (50)		
Occupation					**0.004** ^*∗*^	**14.9**
Unemployed	1 (2)	24 (9)	3 (7)	1 (17)		
Self-employed	23 (56)	133 (52)	33 (83)	4 (66)		
Employed	17 (42)	98 (38)	4 (10)	1 (17)		
Student	0	3 (1)	0			
Marital status					0.487	1.4
. Single	10 (24)	59 (23)	8 (20)	2 (33)		
Married	25 (61)	153 (59)	26 (65)	1 (17)		
Separated	2 (5)	11 (4)	1 (3)	0		
Divorced	0	8 (3)	1 (3)	0		
Widowed	4 (10)	27 (10)	4 (10)	3 (50)		
Smoker					0.529	0.3961
Yes	1 (2)	7 (3)	1 (3)	1 (17)		
No	40 (98)	251 (97)	39 (97)	5 (83)		
Consume alcohol					0.777	0.0803
Yes	4 (10)	26 (10)	4 (10)	0		
No	37 (90)	232 (90)	36 (90)	6 (100)		

**Table 6:** Correlation of baseline medical characteristics and HAND on bivariate analysis.

Variable	HAND category				*p* value	*Χ* ^2^
	Unimpaired *n* (%)	ANI *n* (%)	MND *n* (%)	HAD *n* (%)		
Hypertensive					0.347	0.8846
Yes	4 (10)	37 (14)	4 (10)	0		
No	37 (90)	221 (86)	36 (90)	6 (100)		
Diabetic					0.487	0.4839
Yes	0	13 (5)	1 (2)	0		
No	41 (100)	245 (95)	39 (98)	6 (100)		
CD4 count					0.195	4.7051
<250	6 (15)	60 (24)	8 (20)	0		
250–349	4 (10)	36 (14)	9 (23)	0		
350–499	9 (23)	70 (27)	7 (17)	0		
≥500	21 (52)	89 (35)	16 (40)	6 (100)		
Regimen change					0.392	0.7338
Yes	10 (24)	73 (28)	8 (20)	2 (33)		
No	31 (76)	185 (72)	32 (80)	4 (67)		

**Table 7:** Multivariate logistic regression model for predictors of HAND.

Variables	Coefficients		
	aOR	95% CI	(*p* value)
Female gender	2.17	1.02–4.72	**0.04** ^*∗*^
Education in years	0.58	0.38–0.89	**0.01** ^*∗*^
Years on ART	1.16	0.98–1.38	0.08
Age	1.03	0.99–1.09	0.09
Years with HIV	0.87	0.76–1.01	0.05
CD4	0.998	0.997–0.999	**0.01** ^*∗*^

Increased risk of HAND in women; reduced risk of HAND among those with higher education levels and higher CD4 counts.

**Table 8:** Multivariate logistic regression model for predictors of severe forms of HAND.

Variables	Coefficients		
	aOR	95% CI	(*p* value)
Female gender	5.83	1.25–37.42	**0.04** ^*∗*^
Education in years	0.24	0.08–0.58	**0.003** ^*∗*^
Years on ART	1.29	0.86–2.00	0.24
Age	1.08	1.00–1.17	0.05
PHQ score	7.47	1.69–43.53	**0.01** ^*∗*^
Years with HIV	0.79	0.58–1.04	0.10
CD4	0.998	0.995–1.00	0.13
Hypertension	0.66	0.51–7.26	0.74
Regimen modification	3.69	0.48–3.27	0.22

^*∗*^Reduced risk of HAND in persons with higher education levels; increased risk in women compared to men and those who screen positive for a depressive disorder.

## Data Availability

The data used to support the findings of this study are available from the corresponding author upon request.

## Abbreviations

ANI: Asymptomatic neurocognitive impairment
ART: Antiretroviral therapy
BNCS: Brief neurocognitive screen
CD4: Cluster of differentiation 4
CNS: Central nervous system
COWAT: Controlled Oral Word Association Test
HAART: Highly active antiretroviral therapy
HAD: HIV-associated dementia
HAND: HIV-associated neurocognitive disorders
HIV: Human immunodeficiency virus
IADL: Instrumental activities of daily living
IHDS: International HIV Dementia Scale
KNH: Kenyatta National Hospital
MND: Mild neurocognitive disorder
MOCA: Montreal Cognitive Assessment scale
PLWH: People living with HIV
SD: Standard deviation
WMS-III: Weschler Memory Scale III.
